# Costs associated with failure to respond to treatment among patients with rheumatoid arthritis initiating TNFi therapy: a retrospective claims analysis

**DOI:** 10.1186/s13075-017-1293-1

**Published:** 2017-05-15

**Authors:** Michael Grabner, Natalie N. Boytsov, Qing Huang, Xiang Zhang, Tingjian Yan, Jeffrey R. Curtis

**Affiliations:** 1 HealthCore, Inc., 123 Justison Street, Suite 200, Wilmington, DE 19801 USA; 2 Eli Lilly & Company, Indianapolis, IN USA; 3 Phar LLC, Beverly Hills, CA USA; 4 Division of Clinical Immunology and Rheumatology, University of Alabama at Birmingham School of Medicine, Birmingham, AL USA

**Keywords:** Healthcare resource use, Healthcare costs, Biologic, Real-world observational study, Treatment response, Rheumatoid arthritis

## Abstract

**Background:**

Tumor necrosis factor inhibitors (TNFi) are common second-line treatments for rheumatoid arthritis (RA). This study was designed to compare the real-world clinical and economic outcomes between patients with RA who responded to TNFi therapy and those who did not.

**Methods:**

For this retrospective cohort analysis we used medical and pharmacy claims from members of 14 large U.S. commercial health plans represented in the HealthCore Integrated Research Database. Adult patients (aged ≥18 years) diagnosed with RA and initiating TNFi therapy (index date) between 1 January 2007 and 30 April 2014 were included in the study. Treatment response was assessed using a previously developed and validated claims-based algorithm. Patients classified as treatment responders in the 12 months postindex were matched 1:1 to nonresponders on important baseline characteristics, including sex, age, index TNFi agent, and comorbidities. The matched cohorts were then compared on their all-cause and RA-related healthcare resource use, and costs were assessed from a payer perspective during the first, second, and third years postindex using parametric tests, regressions, and a nonparametric bootstrap.

**Results:**

A total of 7797 patients met the study inclusion criteria, among whom 2337 (30%) were classified as treatment responders. The responders had significantly lower all-cause hospitalizations, emergency department visits, and physical/occupational therapy visits than matched nonresponders during the first-year postindex. Mean total all-cause medical costs were $5737 higher for matched nonresponders, largely driven by outpatient visits and hospitalizations. Mean all-cause pharmacy costs (excluding costs of biologics) were $354 higher for matched nonresponders. Mean RA-related pharmacy costs (conventional synthetic and biologic drugs), however, were $8579 higher in the responder cohort, driven by higher adherence to their index TNFi agent (*p* < 0.01 for all comparisons). A similar pattern of cost differentiation was observed over years 2 and 3 of follow-up.

**Conclusions:**

In this real-world study we found that, compared with matched nonresponders, patients who responded to TNFi treatments had lower all-cause medical, pharmacy, and total costs (excluding biologics) up to 3 years from initiation of TNFi therapy. These cost differences between the two cohorts provide a considerable offset to the cost of RA medications and should encourage close monitoring of treatment response to minimize disease progression with appropriate therapy choices.

**Electronic supplementary material:**

The online version of this article (doi:10.1186/s13075-017-1293-1) contains supplementary material, which is available to authorized users.

## Background

Rheumatoid arthritis (RA) affects a relatively small subset of the American population—an estimated 1.3 million people in 2005 [1]—yet, the condition exerts an outsize impact on total healthcare expenditures as well as on overall quality of life, including lost productivity and early mortality [2]. When direct, indirect, and intangible costs are considered, RA annual costs have been estimated at over $56 billion in the United States (in 2016 U.S. dollars) [3]. Biologic therapies contribute substantially to these costs [4] and should be used as effectively as possible.

Achievement of clinical remission or low disease activity has been shown to reduce pain, prevent joint damage, and preserve functional ability, and is the goal of RA treatment [5–7]. Because the course and severity of RA vary across patients and even for an individual patient over time, treatment regimens should be individualized [8]. Guidelines issued by the American College of Rheumatology in 2015 for the treatment of RA recommend initial treatment with methotrexate (MTX) for patients with established RA, with the addition of another conventional or targeted synthetic or biologic (tumor necrosis factor inhibitors [TNFi] or non-TNFi) disease-modifying antirheumatic drug (DMARD) if symptoms persist at moderate or high disease activity [5]. Treatment changes (i.e., drug addition, switch, or dose changes) are recommended as often as every 3 months, depending on the treatment regimen being used and the change being considered [5].

TNFi agents are often used as the first biologic therapy after patients have an inadequate response or intolerance to MTX [9, 10]. Researchers in a number of studies have evaluated the treatment costs associated with treatment response among TNFi users [11–15]. For example, one recently published U.S. study concluded that the total RA-associated costs in patients with treatment response ranged from $25,086 to $36,107 per patient-year, depending on the specific TNFi agent used [11]. Less is known about cost differences between treatment responders and nonresponders, which is important for evaluating treatment cost-effectiveness. Results of one study suggested that patients in Canada treated with TNFi agents and achieving low disease activity or remission were associated with lower healthcare costs than those with persistent moderate or high disease activity; however, drug costs were not included in this analysis [16]. In a study using Medicare data in the United States, researchers came to a similar conclusion but also did not take drug costs into consideration [17].

The present study was designed to gain a better understanding of the real-world clinical and economic outcomes between patients with RA who responded to TNFi therapies (treatment responders) and those who did not (nonresponders), accounting for medical as well as drug costs. Differences across cohorts would highlight the importance of close monitoring of patients with RA for appropriate treatment adjustments based on their treatment response. Furthermore, this research provides important input on medical cost offsets associated with treatment response for future cost-effectiveness assessments. Using a large administrative claims database, we analyzed differences in healthcare resource utilization (HCRU) and costs between treatment responders and nonresponders, and we explored the potential for cost savings associated with the response. Matching techniques and sensitivity analysis were used to address potential differences in characteristics between the patient cohorts. RA treatment responders were identified using a claims-based algorithm [18]. The algorithm was originally developed to measure the clinical effectiveness of RA treatments and has acceptable performance characteristics for identifying responders based on validation studies using clinical outcomes (e.g., low disease activity/remission or meaningful change in the Disease Activity Score in 28 joints based on erythrocyte sedimentation rate) as the gold standard (positive predictive value 76%, 95% confidence interval (CI) 71% to 81%; negative predictive value 90%, CI 88% to 92%; sensitivity 72%, CI 67% to 77%; specificity 91%, CI 89% to 93%) [18, 19].

## Methods

### Data source

Data for this retrospective longitudinal cohort study were obtained from the HealthCore Integrated Research Database (HIRD®) during the study period of 1 January 2006 through 30 April 2015. The HIRD contains medical and pharmacy administrative claims from approximately 41 million members of 14 commercial health plans geographically distributed across the United States (U.S.). Member enrollment, medical care (professional and facility claims), outpatient prescription drug use, outpatient laboratory test results data, and healthcare costs may be tracked for health plan members in the database dating back to January 2006. Comparisons of the HIRD against U.S. Census data indicate the patient population contained within the HIRD is mostly representative of the general U.S. population [20]. The study was conducted in full compliance with the relevant provisions of the Health Insurance Portability and Accountability Act. All study data were kept anonymous to protect patient confidentiality; researchers had access only to a limited dataset with no patient identifiers. This observational study was conducted under the provisions of Privacy Rule 45 C.F.R. 164.514(e) and was exempt from Institutional Review Board review and approval.

### Study population

Patients were included in the study if they had a medical or pharmacy claim for a TNFi between 1 January 2007 and 30 April 2014 (study intake period). Patients had at least 12 months of continuous health plan enrollment before and after the first claim for a TNFi agent (the index date); patients who died during the follow-up time frame were therefore excluded. All patients were adults (18 years of age or older) as of the index date and diagnosed with RA, identified by at least two outpatient claims or at least one inpatient/emergency department (ED) claim for RA (International Classification of Diseases, Ninth Revision, Clinical Modification [ICD-9-CM], diagnosis codes 714.0x, 714.1x, or 714.2x) at any time during the study period. Patients were excluded if they had a claim for any biologic agent during the 12-month preindex period (baseline). Patients were also excluded if they had one or more claims for comorbid psoriasis or psoriatic arthritis (ICD-9-CM code 696.xx), ankylosing spondylitis (ICD-9-CM code 720.0x), Crohn’s disease (ICD-9-CM code 555.xx), ulcerative colitis (ICD-9-CM code 556.xx), or juvenile polyarthritis (ICD-9-CM code 714.3x) at any time during the study period.

Patients were divided into two mutually exclusive cohorts on the basis of their treatment response during the 12 months after the index date (“responders” and “nonresponders”). Patients were followed up to 3 years after the index date. Following the criteria of the previously published algorithm, we classified patients as treatment responders if they met all of the following six criteria in the 12-month postindex period: (1) an index medication adherence rate of 80% or higher as calculated by proportion of days covered, (2) no switch or addition of any biologic (TNFi and others), (3) no addition of a new conventional synthetic disease-modifying antirheumatic drug (csDMARD), (4) no increase in index TNFi dose or frequency, (5) no more than one glucocorticoid injection, and (6) no increase in dose of oral glucocorticoid treatment [18]. The list of TNFi agents was expanded beyond the original algorithm (which included adalimumab, etanercept, and infliximab) to include all TNFi drugs available on the market at the time of this study (adding certolizumab pegol and golimumab).

### Outcome measures

The primary study outcome measures were all-cause and RA-related HCRU and associated costs during 1 year after the index date. Annual HCRU and associated costs were also reported among patients with 2- or 3-year postindex continuous health plan enrollment. HCRU and costs were examined for inpatient hospitalizations, ED visits, outpatient visits, and pharmacy prescriptions and were reported as all-cause and RA-related (defined by all medical claims with ICD-9-CM diagnoses for RA and all pharmacy claims for DMARDs; DMARDs covered under a medical benefit could not be identified separately). Hospitalizations for joint surgeries, cardiovascular events, and infections were specifically identified. Outpatient visits included physician office visits, physical/occupational therapy visits, imaging (e.g., radiographs, magnetic resonance imaging), laboratory tests, and other visit categories. Pharmacy use included overall prescriptions filled and was also stratified by csDMARDs and biologics. Costs represented health plan-paid amounts and were reported per patient in 2014 U.S. dollars [21].

Patient demographic and clinical characteristics, including the specific index TNFi agent (adalimumab, certolizumab pegol, etanercept, golimumab, infliximab) and specialty of the prescribing provider, RA severity based on the claims-based index for rheumatoid arthritis severity [22], and comorbidities including the Quan-Charlson comorbidity index (QCI) [23], were collected from the index TNFi claim or during the 12-month preindex baseline period.

### Statistical analysis

Descriptive statistics, including means, SDs, and absolute/relative frequencies were reported for continuous and categorical variables, respectively. Patient characteristics were statistically compared between study cohorts. The χ^2^ test was used for categorical variables, and a *t* test or Wilcoxon rank-sum test was used for continuous variables, depending on the variable distributions. Statistical outcomes, such as *p* values and CIs, were reported without multiplicity analysis and should be interpreted accordingly. For all statistical tests, a two-sided 5% significance level was used.

Owing to the observational nature of the study, Mahalanobis matching was used to control for potential differences in baseline characteristics between the two cohorts because the association between baseline covariates and treatment response could confound the association between treatment response and corresponding healthcare costs. A 1:1 nearest neighbor matching algorithm with calipers (equal to 0.2 units of the SD of the Mahalanobis distance) without replacement was used [24]. The matching was performed on the Mahalanobis distance, a singular summary score derived from the following baseline characteristics: sex, age, physician specialty on index claim, index TNFi agent, QCI, mental illness, and any csDMARD use. The postmatching balance of baseline characteristics was assessed by significance testing and assessment of standardized differences of each baseline covariate between cohorts, where absolute standardized differences ≤0.10 indicated an acceptable balance after matching [25].

*t* tests and nonparametric bootstrapping were used for statistical comparisons of observed mean costs between cohorts during the 1-year follow-up period [26]. Among a subset of patients with at least 3 years of available follow-up, a generalized linear mixed model was implemented to measure the trend in cross-cohort cost differences over years 1, 2, and 3 of follow-up. This subgroup was chosen in order to use the same patient cohorts for cost estimation in each of the 3 years. Two mixed models were estimated: one with indicator variables for each of the 3 years and two cohorts (unadjusted), and one that additionally included several baseline patient characteristics (adjusted). Sensitivity analyses on cost differences between responders and nonresponders were performed using regression analysis on the matched cohorts (“double adjustment”) as well as on the full prematch cohorts. Regression analysis allows adjustment for baseline characteristics that were not balanced after matching (in case of the matched cohorts) or not otherwise accounted for (in case of the prematch cohorts). All regressions used generalized linear models with log-link and gamma distribution to adjust for cost data skewness [27] (*see* Additional file 1 for further details on the mixed model as well as the sensitivity analysis).

## Results

### Baseline patient characteristics

Of the 7797 patients who met the study inclusion criteria, 2337 (30%) were treatment responders and 5460 (70%) were nonresponders at 12 months after the index date (*see* Table 1 for details on the algorithm metrics; *see* Additional file 2 for stepwise patient identification results). Prior to matching, responders and nonresponders differed in several characteristics. For example, responders had a lower proportion of female patients (71% vs. 77%), a lower baseline comorbidity burden (QCI 1.5 vs. 1.7), a higher share of etanercept initiators (61% vs. 51%), and a lower share of infliximab users (11% vs. 21%), as well as lower baseline HCRU and costs (*see* Additional file 3).

**Table 1:** Results of the effectiveness algorithm, overall and by index medication

Criterion	Total (*N* = 7797), *n* (%)	Adalimumab (*n* = 1899), *n* (%)	Certolizumab pegol (*n* = 124), *n* (%)	Etanercept (*n* = 4188), *n* (%)	Golimumab (*n* = 170), *n* (%)	Infliximab (*n* = 1416), *n* (%)
Criterion 1: PDC ≥0.8 for index TNFi	3362 (43.1%)	795 (41.9%)	35 (28.2%)	1721 (41.1%)	65 (38.2%)	746 (52.7%)
Criterion 2: Patients with no biologic switch or addition	6286 (80.6%)	1528 (80.5%)	99 (79.8%)	3339 (79.7%)	133 (78.2%)	1187 (83.8%)
Criterion 3: Patients with no addition of a new csDMARD	6705 (86.0%)	1604 (84.5%)	103 (83.1%)	3621 (86.5%)	137 (80.6%)	1240 (87.6%)
Criterion 4: Patients with no increase in index TNFi dose or frequency	7019 (90.0%)	1738 (91.5%)	116 (93.5%)	4151 (99.1%)	170 (100.0%)	844 (59.6%)
Criterion 5: Patients with no more than one glucocorticoid joint injection	6483 (83.1%)	1604 (84.5%)	96 (77.4%)	3591 (85.7%)	140 (82.4%)	1052 (74.3%)
Criterion 6: Patients with no increase in dose of oral glucocorticoid	6907 (88.6%)	1702 (89.6%)	112 (90.3%)	3734 (89.2%)	152 (89.4%)	1207 (85.2%)
Total number of patients with treatment response	2337 (30.0%)	586 (30.9%)	24 (19.4%)	1418 (33.9%)	56 (32.9%)	253 (17.9%)

*Abbreviations: csDMARD* Conventional synthetic disease-modifying antirheumatic drug, *PDC* Proportion of days covered, *TNFi* Tumor necrosis factor inhibitors

Each patient in the responder cohort was successfully matched to a patient in the nonresponder cohort. Following the match, the two cohorts (with *n* = 2337 each) were well-balanced on nearly all characteristics, including sex, age, geographical region, index TNFi, specialty of prescribing physician, baseline HCRU, and costs (*see* Tables 2 and 3). Overall, the mean age of patients included in the study (matched cohorts) was 52 years, and a majority were women (71%). Etanercept was the most commonly prescribed index TNFi (61%), followed by adalimumab (25%) and infliximab (11%). Most prescribing providers were rheumatologists (80%). At baseline, more than 90% of all patients had received csDMARDs, most commonly MTX (79%).

**Table 2:** Baseline demographic and clinical characteristics (postmatching)

	Responders (*n* = 2337)	Nonresponders (*n* = 2337)	*p* Value^a^	Standardized difference^b^
Female sex, *n* (%)	1655 (70.8%)	1659 (71.0%)	0.900	0.00
Age at index date, years, mean (SD)	52.3 (11.30)	52.0 (10.66)	0.466	0.02
Geographic region, *n* (%)				
Northeast	348 (14.9%)	327 (14.0%)	0.382	0.03
South	689 (29.5%)	609 (26.1%)	0.009	0.08
Midwest	731 (31.3%)	786 (33.6%)	0.086	0.05
West	453 (19.4%)	493 (21.1%)	0.145	0.04
Unknown	116 (5.0%)	122 (5.2%)	0.690	0.01
Health plan type, *n* (%)				
HMO	590 (25.2%)	546 (23.4%)	0.133	0.04
PPO	1604 (68.6%)	1642 (70.3%)	0.228	0.04
CDHP	143 (6.1%)	149 (6.4%)	0.717	0.01
Any Medicare plan (Medicare Advantage or Medicare Supplemental plus Part D), *n* (%)	182 (7.8%)	182 (7.8%)	1.000	0.00
TNFi agent on index fill, *n* (%)				
Adalimumab	586 (25.1%)	579 (24.8%)	0.813	0.01
Certolizumab pegol	24 (1.0%)	43 (1.8%)	0.019	0.07
Etanercept	1418 (60.7%)	1418 (60.7%)	1.000	0.00
Golimumab	56 (2.4%)	43 (1.8%)	0.187	0.04
Infliximab	253 (10.8%)	254 (10.9%)	0.962	0.00
Prescribing physician specialty on index TNFi claim, *n* (%)				
Rheumatology	1872 (80.1%)	1864 (79.8%)	0.770	0.01
PCP^c^	35 (1.5%)	38 (1.6%)	0.723	0.01
Other	47 (2.0%)	40 (1.7%)	0.449	0.02
Unknown	383 (16.4%)	395 (16.9%)	0.637	0.01
QCI, mean (SD)	1.5 (1.03)	1.5 (0.99)	0.977	0.00
CIRAS, mean (SD)	6.6 (1.73)	6.5 (1.68)	0.043	0.02
Targeted comorbidities of interest, *n* (%)				
Chronic respiratory/pulmonary conditions	305 (13.1%)	365 (15.6%)	0.012	0.07
CVD^d^	197 (8.4%)	206 (8.8%)	0.592	0.02
Diabetes	280 (12.0%)	250 (10.7%)	0.166	0.04
Dyslipidemia	789 (33.8%)	771 (33.0%)	0.577	0.02
Fibromyalgia	316 (13.5%)	392 (16.8%)	0.002	0.09
Fragility fractures (closed)	34 (1.5%)	37 (1.6%)	0.720	0.01
GI ulcer	23 (1.0%)	26 (1.1%)	0.667	0.01
Hypertension	809 (34.6%)	880 (37.7%)	0.031	0.06
Low-back pain	461 (19.7%)	594 (25.4%)	<0.001	0.14
Mental health issues	476 (20.4%)	471 (20.2%)	0.856	0.01
Osteoarthritis	892 (38.2%)	969 (41.5%)	0.021	0.07
Osteoporosis	231 (9.9%)	254 (10.9%)	0.270	0.03

*Abbreviations: ACS* Acute coronary syndrome, *CDHP* Consumer-driven health plan, *CHD* Coronary heart disease, *CIRAS* Claims-based index for rheumatoid arthritis severity, *CVD* Cardiovascular disease, *GI* Gastrointestinal, *HMO* Health maintenance organization, *MI* Myocardial infarction, *PAD* Peripheral arterial disease, *PCP* Primary care physician, *PPO* Preferred provider organization, *QCI* Quan-Charlson comorbidity index, *TIA* Transient ischemic attack, *TNFi* Tumor necrosis factor inhibitors

^a^ χ^2^ tests were used to determine statistical differences across categorical variables; *t* tests were used for continuous variables

^b^ Standardized difference = difference in means or proportions divided by standard error, in absolute value

^c^ PCP includes family/general practice and internal medicine

^d^ CVD includes ACS (MI and unstable angina), CHD with or without history of MI, ischemic stroke/TIA, PAD, and ventricular arrhythmia

**Table 3:** Baseline all-cause healthcare resource utilization and cost characteristics (postmatching)

	Responders (*n* = 2337)	Nonresponders (*n* = 2337)	*p* Value^a^	Standardized difference^b^
Inpatient hospitalization, *n* (%) with at least one visit	197 (8.4%)	249 (10.7%)	0.010	0.08
ED encounters, *n* (%) with at least one visit	374 (16.0%)	418 (17.9%)	0.086	0.05
Outpatient visits, *n* (%) with at least one visit	2332 (99.8%)	2332 (99.8%)	1.000	0.00
Rheumatologist office visit, *n* (%)	1692 (72.4%)	1623 (69.4%)	0.026	0.07
Physical/occupational therapy visits, *n* (%)	506 (21.7%)	530 (22.7%)	0.398	0.03
Pharmacy fills, *n* (%) with at least one fill	2298 (98.3%)	2309 (98.8%)	0.176	0.04
Oral glucocorticoids	1579 (67.6%)	1699 (72.7%)	<0.001	0.11
Antihypertensives	887 (38.0%)	974 (41.7%)	0.009	0.08
Antidiabetics	206 (8.8%)	184 (7.9%)	0.245	0.03
Antihyperlipidemics	512 (21.9%)	497 (21.3%)	0.594	0.02
Antidepressives	537 (23.0%)	643 (27.5%)	<0.001	0.11
Pain medications^c^	1853 (79.3%)	1891 (80.9%)	0.164	0.04
Number of pharmacy fills per patient, mean (SD)	40.7 (28.11)	43.9 (30.59)	<0.001	0.11
csDMARDs, *n* (%) with at least one fill	2135 (91.4%)	2135 (91.4%)	1.000	0.00
Hydroxychloroquine	701 (30.0%)	658 (28.2%)	0.166	0.04
Leflunomide	301 (12.9%)	322 (13.8%)	0.366	0.03
Methotrexate	1849 (79.1%)	1854 (79.3%)	0.857	0.01
Minocycline	27 (1.2%)	25 (1.1%)	0.780	0.01
Sulfasalazine	266 (11.4%)	270 (11.6%)	0.854	0.01
Total medical costs, $ per person, mean (SD)	6819 (14,807)	7730 (15,929)	0.043	0.06
Inpatient costs, $ per person, mean (SD)	1927 (10,790)	2068 (10,636)	0.653	0.01
ED costs, $ per person, mean (SD)	242 (938)	336 (1527)	0.012	0.07
Outpatient costs, $ per person, mean (SD)	4638 (7152)	5313 (9501)	0.006	0.08
Total pharmacy costs, $ per person, mean (SD)	1639 (2433)	1799 (3020)	0.046	0.06

*csDMARD* Conventional synthetic disease-modifying antirheumatic drug, *ED* Emergency department

^a^ χ^2^ tests were used to determine a statistical differences across categorical variables; *t* tests were used for continuous variables

^b^ Standardized difference = difference in means or proportions divided by standard error, in absolute value

^c^ Pain medications include opioids, nonsteroidal anti-inflammatory drugs, and others, and exclude disease-modifying antirheumatic drugs

### Healthcare resource utilization during follow-up

Treatment responders had generally lower all-cause HCRU than matched nonresponders during the 1-year follow-up period after the index date (*see* Table 4). Specifically, among treatment responders, we observed statistically significantly lower proportions of patients with at least one visit, as well as a lower mean number of visits per patient, in the categories of all-cause inpatient hospitalizations (mean 0.1 vs. 0.2, *p* < 0.01), ED visits (0.1 vs. 0.3, *p* < 0.01), and physical/occupational therapy visits (1.6 vs. 2.3, *p* < 0.01). The proportion of patients with a hospitalization for joint replacement surgery and infection was lower among responders, but it was similar for cardiovascular events. The mean number of overall outpatient visits was also lower among responders (25.8 vs. 32.1, *p* < 0.01), including office visits with rheumatologists (3.1 vs. 3.3, *p* < 0.01).

**Table 4:** All-cause healthcare resource utilization during 1-year follow-up

	Responders (*n* = 2337), *n* (%)/mean (SD)	Nonresponders (*n* = 2337), *n* (%)/Mean (SD)	*p* Value^a^
Inpatient hospitalization, *n* (%) with at least one visit	149 (6.4%)	297 (12.7%)	<0.001
Joint replacement surgeries, *n* (%)	29 (1.2%)	51 (2.2%)	0.013
Infections, *n* (%)	27 (1.2%)	83 (3.6%)	<0.001
CV events, *n* (%)	24 (1.0%)	25 (1.1%)	0.886
ED encounters, *n* (%) with at least one visit	278 (11.9%)	461 (19.7%)	<0.001
Outpatient visits, *n* (%) with at least one visit	2334 (99.9%)	2326 (99.5%)	0.032
Physician office visit, *n* (%)	2329 (99.7%)	2324 (99.4%)	0.274
Rheumatologist office visit, *n* (%)	1706 (73.0%)	1638 (70.1%)	0.027
Physical/occupational therapy visits, *n* (%)	390 (16.7%)	512 (21.9%)	<0.001
DME claims, *n* (%)	400 (17.1%)	578 (24.7%)	<0.001
Imaging^b^ claims, *n* (%)	1676 (71.7%)	1803 (77.2%)	<0.001
Pharmacy fills, *n* (%) with at least one fill	2330 (99.7%)	2331 (99.7%)	0.781
Oral glucocorticoids	1040 (44.5%)	1463 (62.6%)	<0.001
Antihypertensives	919 (39.3%)	1017 (43.5%)	0.004
Antidiabetics	208 (8.9%)	201 (8.6%)	0.717
Antihyperlipidemics	525 (22.5%)	524 (22.4%)	0.972
Antidepressives	541 (23.1%)	700 (30.0%)	<0.001
Pain medications^c^	1542 (66.0%)	1739 (74.4%)	<0.001
Number of pharmacy fills per patient, mean (SD)	49.4 (29.44)	50.9 (33.12)	0.109
csDMARDs, *n* (%) with at least one fill	1945 (83.2%)	1937 (82.9%)	0.755
Hydroxychloroquine	504 (21.6%)	538 (23.0%)	0.232
Leflunomide	219 (9.4%)	277 (11.9%)	0.006
Methotrexate	1607 (68.8%)	1596 (68.3%)	0.729
Minocycline	18 (0.8%)	20 (0.9%)	0.745
Sulfasalazine	152 (6.5%)	173 (7.4%)	0.227
Biologics, *n* (%) with at least one fill	2080 (89.0%)	2088 (89.3%)	0.706
Adalimumab	586 (25.1%)	840 (35.9%)	<0.001
Certolizumab pegol	20 (0.9%)	65 (2.8%)	<0.001
Etanercept	1418 (60.7%)	1512 (64.7%)	0.004
Golimumab	48 (2.1%)	98 (4.2%)	<0.001
Infliximab	8 (0.3%)	14 (0.6%)	0.200
Abatacept	0 (0%)	42 (1.8%)	<0.001
Anakinra	0 (0%)	3 (0.1%)	0.083
Rituximab	0 (0%)	2 (0.1%)	0.157
Tocilizumab	0 (0%)	7 (0.3%)	0.008
Tofacitinib citrate	0 (0%)	13 (0.6%)	<0.001
Number of csDMARD pharmacy fills per patient, mean (SD)	7.8 (6.14)	7.1 (5.96)	<0.001
Number of biologic pharmacy fills per patient, mean (SD)	9.5 (4.62)	6.1 (4.25)	<0.001

*Abbreviations: ACS* Acute coronary syndrome, *csDMARD* Conventional synthetic disease-modifying antirheumatic drug, *CV* Cardiovascular, *DME* Durable medical equipment, *ED* Emergency department, *RA* Rheumatoid arthritis, *TIA* Transient ischemic attack

Joint replacement surgeries include knee, hip, shoulder, and other surgeries. CV events include ACS, coronary revascularization, ischemic stroke, and TIA

^a^ χ^2^ tests were used to determine statistical differences across categorical variables; *t* tests were used for continuous variables

^b^ Imaging included radiographs, magnetic resonance imaging, and other types of imaging

^c^ Pain medications include opioids, nonsteroidal anti-inflammatory drugs, and others, and exclude disease-modifying antirheumatic drugs

The proportion of patients with any prescription fills was similar between the two cohorts. Some medication classes (oral glucocorticoids, antihypertensives, antidepressants, and pain medications) had a lower proportion of responders using them. (Use of these medications was slightly lower among responders at baseline as well; in all cases, the gap between the cohorts increased during follow-up.) The cohorts also had a similar mean number of all-cause prescription fills per patient (49.4 vs. 50.9, *p* = 0.109). For RA-related medications, however, the responders had more prescription fills than nonresponders overall (17.4 vs. 13.1, *p* < 0.001), as well as for csDMARDs and biologics separately.

### Healthcare costs during follow-up

**Fig. 1:**
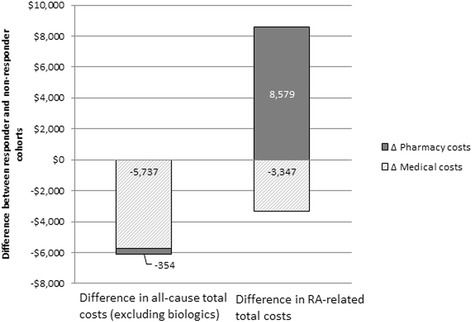
Cost differences between responders and nonresponders during 1 year follow-up (*n* = 2337 per cohort). All-cause medical costs include all costs related to inpatient and outpatient visits, such as office visits and laboratory testing. All-cause pharmacy costs include all costs related to outpatient pharmacy fills, with the exception of fills for biologic drugs (defined as abatacept, adalimumab, anakinra, certolizumab pegol, etanercept, golimumab, infliximab, rituximab, tocilizumab, and tofacitinib citrate). Rheumatoid arthritis (RA)-related medical costs include all costs related to inpatient and outpatient visits, such as office visits and laboratory testing, with International Classification of Diseases, Ninth Revision, Clinical Modification, codes for RA noted on the claims. RA-related pharmacy costs include all costs related to outpatient pharmacy fills for conventional synthetic disease-modifying antirheumatic drugs and biologics

**Fig. 2:**
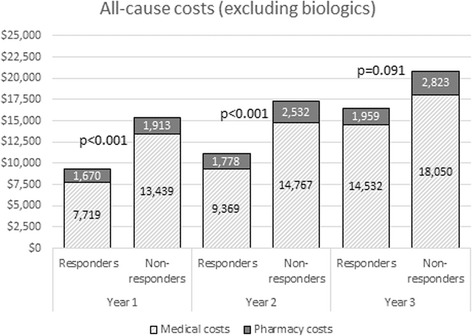
All-cause total healthcare costs (excluding biologics) during 1, 2, and 3 years of follow-up (*n* = 542 per cohort). Costs are derived from a matched sample of patients with ≥3 years of continuous health plan enrollment from the index date (*n* = 542 per cohort). *p* values are derived from *t* tests comparing mean costs across cohorts within each year. Medical costs include all costs related to inpatient and outpatient visits, such as office visits and laboratory testing. Pharmacy costs include all costs related to outpatient pharmacy fills, with the exception of fills for biologic drugs (defined as abatacept, adalimumab, anakinra, certolizumab pegol, etanercept, golimumab, infliximab, rituximab, tocilizumab, and tofacitinib citrate)

All-cause medical costs and all-cause pharmacy costs (excluding biologics) were lower among responders than nonresponders during the first year of follow-up (*see* Table 5 and Fig. 1). Specifically, mean total medical costs per patient were $5737 lower among responders (*p* < 0.01), largely driven by lower outpatient visit costs of $3702 and lower inpatient hospitalization costs of $1755. The majority of these medical costs (59%) were RA-related. All-cause mean total (medical plus pharmacy) costs (excluding biologics) were $6092 lower among responders. A similar pattern was observed over years 2 and 3 of follow-up (*see* Fig. 2).

**Table 5:** All-cause and rheumatoid arthritis-related healthcare costs during 1-year follow-up

Healthcare costs^a^ per patient	Responders (*n* = 2337)		Nonresponders (*n* = 2337)		*p* Value^b^
	Mean (SD)	Median (range)	Mean (SD)	Median (range)	
Medical, all-cause					
Total medical	7581 (15,575)	2123 (0 – 301,318)	13,318 (23,433)	4238 (0 – 337,588)	<0.001
Inpatient hospitalizations	1509 (9,676)	0 (0 – 239,362)	3264 (14,549)	0 (0 – 314,893)	<0.001
Joint replacement surgeries	338 (3415)	0 (0 - 77,382)	656 (5403)	0 (0 - 102,929)	0.016
Infections	149 (2154)	0 (0 - 81,742)	826 (7412)	0 (0 - 225,407)	<0.001
CV events	277 (6198)	0 (0 - 239,362)	464 (6482)	0 (0 - 211,055)	0.312
ED encounters	207 (962)	0 (0 – 16,039)	460 (1909)	0 (0 – 40,264)	<0.001
Outpatient visits	5863 (10,666)	1922 (0 – 148,294)	9565 (15,840)	3460 (0 – 252,428)	<0.001
Physician office visits	1066 (877)	863 (0 – 15,573)	1424 (1237)	1118 (0 – 13,152)	<0.001
Rheumatologist office visits	304 (342)	221 (0 – 3715)	357 (455)	232 (0 – 4284)	<0.001
Physical/occupational therapy	131 (534)	0 (0 - 7567)	222 (851)	0 (0 - 14,319)	<0.001
DME	393 (10,188)	0 (0 - 484,558)	486 (4609)	0 (0 - 122,724)	0.686
Imaging^c^	930 (2596)	210 (0 – 46,467)	1559 (3971)	350 (0 – 48,412)	<0.001
Pharmacy, all-cause (excluding biologics)	1698 (2728)	903 (0 – 22,905)	2052 (3370)	1004 (0 – 41,748)	<0.001
Total, all-cause (medical plus pharmacy, excluding biologics)	9278 (16,142)	3919 (0 – 306,913)	15,370 (24,447)	6599 (0 – 348,118)	<0.001
Medical, RA-related	4498 (11,150)	590 (0 - 141,903)	7845 (16,300)	889 (0 - 282,902)	<0.001
Pharmacy, RA-related	21,852 (9572)	23,042 (0 – 77,237)	13,273 (10,131)	12,445 (0 – 59,331)	<0.001
csDMARDs	214 (376)	87 (0 – 6992)	181 (360)	64 (0 – 6649)	0.002
Biologics	21,808 (9719)	22,979 (0 – 77,220)	13,193 (10,212)	12,337 (0 – 68,752)	<0.001
Total, RA-related (medical and pharmacy)	26,350 (10,196)	24,668 (191 – 142,091)	21,118 (16,725)	18,716 (0 – 285,900)	<0.001
Total, all-cause (medical plus pharmacy, including biologics)	31,087 (15,556)	28,268 (2017 - 332,724)	28,563 (24,600)	23,701 (0 - 350,986)	<0.001

*Abbreviations: csDMARD* Conventional synthetic disease-modifying antirheumatic drug, *CV* Cardiovascular, *DME* Durable medical equipment, *ED* Emergency department, *RA* Rheumatoid arthritis

^a^ Plan-paid costs in 2014 U.S. dollars, assessed over the first year postindex

^b^ χ^2^ tests were used to determine statistical differences across categorical variables; *t* tests were used for continuous variables. In addition, a nonparametric bootstrap was used to calculate a second set of *p* values for comparisons of key mean cost metrics; these *p* values were similar to the ones derived from the *t* tests. The table therefore reports the *t* test *p* values

^c^ Imaging included radiographs, magnetic resonance imaging, and other types of imaging

**Fig. 3:**
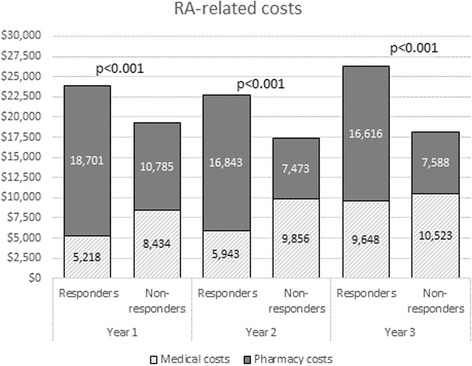
Rheumatoid arthritis (RA)-related total healthcare costs during 1, 2, and 3 years of follow-up (*n* = 542). Costs are derived from a matched sample of patients with ≥3 years of continuous health plan enrollment from the index date (*n* = 542 per cohort). *p* values are derived from *t* tests comparing mean costs across cohorts within each year. Medical costs include all costs related to inpatient and outpatient visits, such as office visits and laboratory testing, with International Classification of Diseases, Ninth Revision, Clinical Modification, codes for RA noted on the claims. Pharmacy costs include all costs related to outpatient pharmacy fills for conventional synthetic disease-modifying antirheumatic drugs and biologics

Whereas mean total all-cause pharmacy costs (excluding biologics) were $354 lower among responders than among nonresponders, total mean RA-related pharmacy costs (csDMARDs and biologics) were $8579 higher for responders during the first year of follow-up (*see* Table 5 and Fig. 1). Biologics accounted for the bulk of the cost difference (mean per-patient cost $21,808 vs. $13,193, *p* < 0.01). The higher RA-related mean total medical and pharmacy costs among responders were driven by the high RA-related pharmacy costs (specifically, the cost of biologics). However, nonresponders had higher mean RA-related medical costs. This pattern was consistent over all 3 years of follow-up (*see* Fig. 3).

Sensitivity analysis using multivariable regression among the matched cohorts showed that nonresponders had 22% higher all-cause pharmacy costs (excluding biologics), 96% higher all-cause medical costs, and 73% higher all-cause total costs during the first year of follow-up, which is consistent with the descriptive results shown in Table 5. A similar sensitivity analysis result was observed using regression analysis of all-cause medical costs in the full prematch sample (*see* Additional file 1 for detailed results).

Results from both unadjusted and adjusted mixed models also suggested that all-cause mean medical and pharmacy costs (excluding biologics) increased over time (*p* < 0.01) in both cohorts, by approximately 24% year-on-year. Furthermore, compared with responders, nonresponders had higher mean costs in every year (*p* < 0.01). These cross-cohort differences remained consistent over time. (The *p* value of the interaction term between cohorts and time in the generalized linear mixed model was >0.9.)

## Discussion

Results of this real-world analysis indicate that patients with RA initiating treatment with TNFi who responded to treatment had consistently lower medical HCRU, medical costs, and nonbiologic pharmacy costs than those who did not respond. These findings were observed over the first year of follow-up (during which some of the costs were related to achievement of the treatment response) as well as over the second and third years of follow-up (the downstream costs of maintaining/failing to maintain treatment response). Comprehensive matching and regression adjustments were performed to account for observed differences between cohorts at baseline. Robustness of the results was confirmed by the sensitivity analysis.

The algorithm for treatment response required high adherence to the index TNFi, resulting in higher biologic therapy pharmacy costs in responders, which was observed in all 3 years. The reductions in medical costs may be considered as the cost offset for this investment, particularly in years 2 and 3 of the analysis. Compared with nonresponders, RA-related mean pharmacy costs were $8579 higher among responders, whereas all-cause mean medical and pharmacy costs (excluding biologics) were $6092 lower, achieving a cost offset of 71%. Our results do not necessarily reflect rebates and discounts on medications that payers may receive from manufacturers. Such discounts would be expected to increase the value of the cost offset and potentially be cost-saving overall. At a 30% discount, for example, biologics would be cost-saving, and the total costs in the responder cohort would be less than those in the nonresponder cohort. In the future, perhaps newer therapies and the availability of biosimilars might make this magnitude of discounting attainable in some settings. Our analysis also considered all TNFi therapies in the aggregate; estimating cost differences across responders and nonresponders specific to each TNFi may be a valuable target for future research.

From a payer perspective, biologic therapy acquisition costs and effective use are crucial elements in the successful management of care for these patients. Quantitative information about the cost savings associated with treatment response, as described in our study, allows informed decision-making on the part of payers. For example, there may be patients who initiate a new drug and are kept on it despite little progress, with the therapy eventually failing. For such nonresponders with high adherence (accounting for 1025 patients [19%] among the 5460 nonresponders identified in our study), resources may be used more efficiently in other ways, and our study provides an estimate of the cost savings associated with response. Similar opportunities for cost savings may exist in situations where patients do not respond well to their initial treatment and are switched to a different treatment, but not in a timely manner, or where existing treatments are titrated with little clinical benefit or cost savings as compared with switching. Health plans may consider aligning their preauthorization processes in such a way as to facilitate switching across different biologic therapies to accelerate patients’ getting a treatment to which they respond. From a physician’s perspective, our study findings also reinforce the benefits of close monitoring of patients’ treatment responses and demonstrate the potential costs of clinical inertia.

Mean medical costs increased over the 3 years of follow-up in both cohorts, possibly owing to disease progression over time. At the same time, pharmacy costs—particularly for biologics—decreased slightly during the follow-up period, perhaps partly because of treatment discontinuation. It is important to note that the study sample used for the 3-year analysis included only those patients who had continuous health plan enrollment for at least 3 years. Costs may have been different for these patients than for those who switched to different types of health plans. Furthermore, patients who were initially responding may have had no response in later years (and vice versa), because the algorithm was implemented only in the first year after TNFi initiation.

Our findings add to previous claims-based research using the same validated algorithm to evaluate treatment effectiveness. The results produced by using the algorithm in the HIRD are consistent with results in other datasets; that is, approximately 30% of patients initiating biologics respond adequately to the treatment [12–14, 18, 19]. Although this proportion may appear low, it is consistent with an American College of Rheumatology 50% improvement metric rate in an MTX- or TNF-inadequate response population participating in large RA clinical trials [28].

Most previous studies have been focused on assessing the costs of specific biologic therapies among responding patients rather than on comparing them with nonresponders. For example, two algorithm-based analyses of patients responding to TNFi therapy concluded that etanercept, which was also the most commonly used medication in our study, had the lowest disease-related medical plus pharmacy cost per patient-year in response [11], as well as the lowest average cost per effectively treated patient [14]. In a separate study of drug costs over 1 year in responders, researchers found that the highest effectiveness rates were among patients using etanercept and adalimumab, and that subcutaneous TNFi agents were more effective and less costly than intravenous biologics [15]. This may be due to the high costs associated with potential dose escalation of an intravenous therapy such as infliximab. Researchers in two studies compared economic outcomes across responders and nonresponders. In a Canadian study assessing response by disease activity measures, Barnabe and colleagues showed economic benefits for patients achieving remission or low disease activity with TNFi treatment, although drug costs were not included in their study [16]. A similar finding was reported by authors of a recent analysis linking lower RA disease activity to reduced medical costs in a Medicare population [17]. Our study extends these results by accounting for DMARD costs, looking at trends over time, and using a large, real-world U.S. patient population.

Our results nevertheless should be interpreted in light of some limitations. The algorithm used for assessing response did not originally include certolizumab pegol and golimumab. It also has not been validated in the HIRD; however, it has been validated in another similar claims database and used in several others [12, 13, 15, 19]. Patients’ discontinuation was the primary factor in defining treatment nonresponse; however, patients might discontinue a therapy for nonclinical reasons as well, such as financial burden. All patients were members of a large U.S. commercial health plan, and the results may not be generalizable to patients with other types of insurance or to those living outside the U.S. Although not fully generalizable, comparisons of the HIRD against U.S. Census data indicate the patient population contained within the HIRD is mostly representative of the general U.S. population. There are some differences with respect to geographic regions and age profiles; for example, the HIRD overrepresents ages between 30 and 64 years and underrepresents ages 65+ years [20]. Costs were obtained from one health plan and may not represent costs incurred by other health plans, nor may they reflect purchasing discounts or rebates. Biologic drugs administered under the plan’s medical benefit are not consistently identifiable in the claims; our reported drug costs are focused on treatments administered under the pharmacy benefit and may underestimate the full biologic cost burden. Our analysis included patients who initiated TNFi agents between 2007 and 2014, with approximately 53% of patients initiating between 2010 and 2014. RA treatment patterns are rapidly evolving, and our results are limited to observed patterns during this time frame. Last, use of claims data limits the ability to assess certain risk factors (e.g., weight, RA disease severity) that may influence treatment response and costs.

## Conclusions

Findings derived from this real-world analysis suggest that patients with treatment response, compared to patients without response, had lower all-cause medical, pharmacy, and total costs (excluding biologics) over up to 3 years from initiation of TNFi therapy. Responders also had lower RA-related medical and overall HCRU and costs. However, RA-related pharmacy costs among responders were higher than among nonresponders, owing to higher adherence to the index biologic therapy. Findings derived from our trend analysis further suggest that the increased costs among nonresponders were consistent over time and that the differences across cohorts remained unchanged. Differences in HCRU and medical costs between the two cohorts represent an offset to the cost of RA treatment and should encourage close monitoring of treatment response to minimize disease progression with appropriate therapy choices. Future researchers may consider examining the presence of such cost offsets for other RA treatments (e.g., non-TNFi biologics). Health plan decision-making may also be improved when taking treatment response based on available claims data into account, and facilitating switching, especially in light of the forthcoming biosimilar medications, where cost will be a driving consideration.

## Additional files

Additional file 1: Supplementary information on methods and regression results. (DOCX 26 kb)

Additional file 2: Patient identification results. (DOCX 15 kb)

Additional file 3: Patient characteristics and baseline HCRU/costs before matching. (DOCX 21 kb)

## Abbreviations

ACS: Acute coronary syndrome
CDHP: Consumer-driven health plan
CHD: Coronary heart disease
CIRAS: Claims-based index for rheumatoid arthritis severity
csDMARD: Conventional synthetic disease-modifying antirheumatic drug
CV: Cardiovascular
CVD: Cardiovascular disease
DMARD: Disease-modifying antirheumatic drug
DME: Durable medical equipment
ED: Emergency department
GI: Gastrointestinal
HCRU: Healthcare resource utilization
HIRD: HealthCore Integrated Research Database
HMO: Health maintenance organization
ICD-9-CM: International Classification of Diseases, Ninth Revision, Clinical Modification
MI: Myocardial infarction
MTX: Methotrexate
PAD: Peripheral arterial disease
PCP: Primary care physician
PDC: Proportion of days covered
PPO: Preferred provider organization
QCI: Quan-Charlson comorbidity index
RA: Rheumatoid arthritis
TIA: Transient ischemic attack
TNFi: Tumor necrosis factor inhibitors
