# Supplementary material for: Costs associated with failure to respond to treatment among patients with rheumatoid arthritis initiating TNFi therapy: a retrospective claims analysis
Source: Arthritis Res Ther. 2017 May 15;19:92. doi: 10.1186/s13075-017-1293-1 (PMC5433023; doi:10.1186/s13075-017-1293-1)
Supplement: Supplementary file 1 — Supplementary information on methods and regression results. (DOCX 26 kb) [file 13075_2017_1293_MOESM1_ESM.docx]

**Supplementary information on methods and regression results**

Generalized linear mixed model

Random effects were modelled through the intercept. Fixed effects differed across the two models: the simple model included flags for each of the 3 years and 2 cohorts, and their interaction; the augmented model further added the following baseline characteristics: age on index date, gender, having adalimumab or infliximab prescription fills on index date, index physician specialty, having a diagnosis for mental illness or for low back pain, using oral glucocorticoids, antidepressant drugs, or cDMARDs, QCI score, all-cause inpatient length of stay, number of all-cause total pharmacy prescription fills, number of antidepressant prescription fills, number of pain medication fills, number of opioid fills, number of cDMARD prescription fills, number of MTX prescription fills, all-cause medical plan-paid costs and all-cause pharmacy plan-paid costs.

List of variables used in regression models

The multivariable model included the following variables:

- All variables used in the matching process
- All baseline variables with absolute SMD>0.10 after matching
- Baseline costs

The full list is as follows:

- Categorical
  - Female
  - Etanercept as index drug
  - Infliximab as index drug
  - Prescribing/treating physician specialty of PCP
  - Prescribing/treating physician specialty of “Other”
  - Flag for mental illness
  - Flag for low-back pain
  - Flag for any glucosteroid (pharmacy) use
  - Flag for any antidepressant use
  - Flag for any opioid use
  - Flag for any csDMARD use
- Continuous
  - Age
  - Quan-Charlson Comorbidity Index score
  - Sum of length of stay across all-cause inpatient hospitalizations
  - Number of all pharmacy fills
  - Number of fills for any antidepressant
  - Number of fills for any pain medication
  - Number of fills for any opioid use
  - Number of fills for any csDMARD
  - Number of fills for methotrexate
  - All-cause plan-paid medical costs
  - All-cause plan-paid pharmacy costs

Results of the regression analysis for the pre-match samples (medical costs only)

| ***All-cause total medical costs over 1-year follow-up (excluding biologics)*** | | | | |
| --- | --- | --- | --- | --- |
| **Covariate** | **Cost ratio^a^** | **95% CI LL** | **95% CI UL** | **p-Value** |
| Cohort (1=responders) | 1.9121 | 1.8048 | 2.0258 | <.0001 |
| Gender (1=female) | 1.0012 | 0.9417 | 1.0645 | 0.9691 |
| Etancercept as index drug | 0.9866 | 0.9286 | 1.0482 | 0.6626 |
| Infliximab as index drug | 3.0413 | 2.8043 | 3.2983 | <.0001 |
| PCP specialty | 0.9521 | 0.8994 | 1.0078 | 0.0908 |
| Other specialty | 1.1775 | 1.057 | 1.3117 | 0.003 |
| Mental illness | 1.0718 | 1.0038 | 1.1444 | 0.0383 |
| Low-back pain | 1.1921 | 1.1207 | 1.268 | <.0001 |
| Rx glucocorticoid use (1=yes) | 1.0081 | 0.9499 | 1.07 | 0.7897 |
| Antidrepressant use (1=yes) | 1.1033 | 1.0088 | 1.2067 | 0.0315 |
| Opioid use (1=yes) | 1.1275 | 1.0592 | 1.2002 | 0.0002 |
| csDMARD use (1=yes) | 0.9813 | 0.8951 | 1.0759 | 0.6879 |
| Age | 1.0012 | 0.9417 | 1.0645 | <.0001 |
| Quan-Charlson Comorbidity Index score | 0.9850 | 0.9752 | 0.9948 | <.0001 |
| Length of stay from all-cause hospitalizations | 1.0037 | 1.0021 | 1.0053 | 0.0029 |
| Number of all pharmacy fills | 0.9893 | 0.9805 | 0.9981 | <.0001 |
| Number of fills for any antidepressant | 0.9989 | 0.9924 | 1.0053 | 0.0172 |
| Number of fills for any pain medication | 1.0050 | 0.9964 | 1.0137 | 0.7314 |
| Number of fills for any opioid use | 1.0002 | 0.9939 | 1.0066 | 0.2568 |
| Number of fills for any csDMARD | 0.9829 | 0.9739 | 0.9919 | 0.9409 |
| Number of fills for methotrexate | 1.0143 | 1.0121 | 1.0166 | 0.0002 |
| All-cause plan-paid medical costs | 1.0200 | 1.0092 | 1.0309 | <.0001 |
| All-cause plan-paid pharmacy costs | 2.1128 | 2.0711 | 2.1565 | 0.0002 |
| CI=confidence interval; LL=lower limit; UL=upper limit; csDMARD=conventional synthetic disease modifying antirheumatic drug; PCP=Primary care physician  ^a^Cost ratios are exponentiated coefficients from generalized linear model regressions with log link and gamma distribution, and can be interpreted like odds ratios. | | | | |

Results of the regression analysis for the matched samples (medical, Rx, and combined costs)

| ***All-cause medical costs over 1-year follow-up (excluding biologics)*** | | | | |
| --- | --- | --- | --- | --- |
| **Covariate** | **Cost ratio^a^** | **95% CI LL** | **95% CI UL** | **p-Value** |
| Cohort (1=responders) | 1.9555 | 1.8243 | 2.0961 | <.0001 |
| Gender (1=female) | 1.0063 | 0.9306 | 1.088 | 0.8758 |
| Etancercept as index drug | 0.9449 | 0.8739 | 1.0216 | 0.1547 |
| Infliximab as index drug | 3.8558 | 3.4021 | 4.37 | <.0001 |
| PCP specialty | 0.9373 | 0.8704 | 1.0093 | 0.0863 |
| Other specialty | 1.1471 | 0.9731 | 1.3522 | 0.1019 |
| Mental illness | 1.0593 | 0.9646 | 1.1632 | 0.2279 |
| Low-back pain | 1.2301 | 1.1296 | 1.3396 | <.0001 |
| Rx glucocorticoid use (1=yes) | 0.9803 | 0.9063 | 1.0605 | 0.6204 |
| Antidrepressant use (1=yes) | 1.1466 | 1.0115 | 1.2997 | 0.0324 |
| Opioid use (1=yes) | 1.1201 | 1.0323 | 1.2154 | 0.0064 |
| csDMARD use (1=yes) | 1.1138 | 0.9717 | 1.2767 | 0.1216 |
| Age | 1.0121 | 1.0086 | 1.0154 | <.0001 |
| Quan-Charlson Comorbidity Index score | 1.0295 | 0.9893 | 1.0714 | 0.1533 |
| Length of stay from all-cause hospitalizations | 0.9689 | 0.9506 | 0.9875 | 0.0011 |
| Number of all pharmacy fills | 1.0072 | 1.0049 | 1.0094 | <.0001 |
| Number of fills for any antidepressant | 0.9843 | 0.9719 | 0.9969 | 0.0145 |
| Number of fills for any pain medication | 1.0023 | 0.9936 | 1.0111 | 0.6104 |
| Number of fills for any opioid use | 0.9905 | 0.9792 | 1.0019 | 0.1037 |
| Number of fills for any csDMARD | 0.9965 | 0.9883 | 1.0047 | 0.4005 |
| Number of fills for methotrexate | 0.9726 | 0.9610 | 0.9842 | <.0001 |
| All-cause plan-paid medical costs | 1.0201 | 1.0162 | 1.0241 | <.0001 |
| All-cause plan-paid pharmacy costs | 1.0132 | 0.9969 | 1.0297 | 0.1128 |
| CI=confidence interval; LL=lower limit; UL=upper limit; csDMARD=conventional synthetic disease modifying antirheumatic drug; PCP=Primary care physician  ^a^Cost ratios are exponentiated coefficients from generalized linear model regressions with log link and gamma distribution, and can be interpreted like odds ratios. | | | | |

| ***All-cause pharmacy costs over 1-year follow-up (excluding biologics)*** | | | | |
| --- | --- | --- | --- | --- |
| **Covariate** | **Cost ratio^a^** | **95% CI LL** | **95% CI UL** | **p-Value** |
| Cohort (1=responders) | 1.2174 | 1.1392 | 1.301 | <.0001 |
| Gender (1=female) | 1.0923 | 1.0138 | 1.1768 | 0.0204 |
| Etancercept as index drug | 0.5984 | 0.5548 | 0.6454 | <.0001 |
| Infliximab as index drug | 0.787 | 0.6965 | 0.8892 | 0.0001 |
| PCP specialty | 1.0578 | 0.9852 | 1.1358 | 0.1216 |
| Other specialty | 1.0146 | 0.8655 | 1.1894 | 0.8581 |
| Mental illness | 1.005 | 0.9184 | 1.0997 | 0.9139 |
| Low-back pain | 1.0271 | 0.9472 | 1.1137 | 0.5177 |
| Rx glucocorticoid use (1=yes) | 0.9492 | 0.8799 | 1.024 | 0.1782 |
| Antidrepressant use (1=yes) | 1.0336 | 0.9165 | 1.1655 | 0.5902 |
| Opioid use (1=yes) | 0.9046 | 0.8362 | 0.9787 | 0.0125 |
| csDMARD use (1=yes) | 1.0784 | 0.9414 | 1.2354 | 0.2761 |
| Age | 1.0027 | 0.9993 | 1.0061 | 0.12 |
| Quan-Charlson Comorbidity Index score | 1.0839 | 1.0425 | 1.1269 | <.0001 |
| Length of stay from all-cause hospitalizations | 0.9704 | 0.9493 | 0.9919 | 0.0074 |
| Number of all pharmacy fills | 1.0066 | 1.0044 | 1.0088 | <.0001 |
| Number of fills for any antidepressant | 1.0052 | 0.9933 | 1.0172 | 0.3927 |
| Number of fills for any pain medication | 1.0000 | 0.9918 | 1.0083 | 0.9979 |
| Number of fills for any opioid use | 1.0062 | 0.9951 | 1.0173 | 0.2735 |
| Number of fills for any csDMARD | 0.9973 | 0.9897 | 1.0049 | 0.4832 |
| Number of fills for methotrexate | 0.9750 | 0.9644 | 0.9859 | <.0001 |
| All-cause plan-paid medical costs | 1.0016 | 0.9983 | 1.0049 | 0.331 |
| All-cause plan-paid pharmacy costs | 1.2517 | 1.2246 | 1.2793 | <.0001 |
| CI=confidence interval; LL=lower limit; UL=upper limit; csDMARD=conventional synthetic disease modifying antirheumatic drug; PCP=Primary care physician  ^a^Cost ratios are exponentiated coefficients from generalized linear model regressions with log link and gamma distribution, and can be interpreted like odds ratios. | | | | |

| ***All-cause total medical plus pharmacy costs over 1-year follow-up (excluding biologics)*** | | | | |
| --- | --- | --- | --- | --- |
| **Covariate** | **Cost ratio^a^** | **95% CI LL** | **95% CI UL** | **p-Value** |
| Cohort (1=responders) | 1.7262 | 1.6249 | 1.8339 | <.0001 |
| Gender (1=female) | 1.0248 | 0.9575 | 1.0969 | 0.4792 |
| Etancercept as index drug | 0.8602 | 0.8035 | 0.921 | <.0001 |
| Infliximab as index drug | 3.0981 | 2.7752 | 3.4585 | <.0001 |
| PCP specialty | 0.9634 | 0.9031 | 1.0278 | 0.2586 |
| Other specialty | 1.1435 | 0.99 | 1.321 | 0.0683 |
| Mental illness | 1.0551 | 0.9719 | 1.1455 | 0.2004 |
| Low-back pain | 1.189 | 1.1034 | 1.2813 | <.0001 |
| Rx glucocorticoid use (1=yes) | 0.9719 | 0.9074 | 1.0409 | 0.415 |
| Antidrepressant use (1=yes) | 1.1196 | 1.0032 | 1.2496 | 0.0437 |
| Opioid use (1=yes) | 1.1024 | 1.0266 | 1.1839 | 0.0074 |
| csDMARD use (1=yes) | 1.11 | 0.9854 | 1.2504 | 0.0859 |
| Age | 1.0101 | 1.0070 | 1.0130 | <.0001 |
| Quan-Charlson Comorbidity Index score | 1.0460 | 1.0096 | 1.0836 | 0.0126 |
| Length of stay from all-cause hospitalizations | 0.9700 | 0.9536 | 0.9866 | 0.0004 |
| Number of all pharmacy fills | 1.0070 | 1.0050 | 1.0090 | <.0001 |
| Number of fills for any antidepressant | 0.9887 | 0.9778 | 0.9996 | 0.043 |
| Number of fills for any pain medication | 1.0015 | 0.9939 | 1.0091 | 0.7029 |
| Number of fills for any opioid use | 0.9916 | 0.9818 | 1.0017 | 0.1046 |
| Number of fills for any csDMARD | 0.9971 | 0.9900 | 1.0043 | 0.4249 |
| Number of fills for methotrexate | 0.9736 | 0.9635 | 0.9837 | <.0001 |
| All-cause plan-paid medical costs | 1.0165 | 1.0133 | 1.0199 | <.0001 |
| All-cause plan-paid pharmacy costs | 1.0598 | 1.0432 | 1.0767 | <.0001 |
| CI=confidence interval; LL=lower limit; UL=upper limit; csDMARD=conventional synthetic disease modifying antirheumatic drug; PCP=Primary care physician  ^a^Cost ratios are exponentiated coefficients from generalized linear model regressions with log link and gamma distribution, and can be interpreted like odds ratios. | | | | |
