# Supplementary material for: Costs associated with failure to respond to treatment among patients with rheumatoid arthritis initiating TNFi therapy: a retrospective claims analysis
Source: Arthritis Res Ther. 2017 May 15;19:92. doi: 10.1186/s13075-017-1293-1 (PMC5433023; doi:10.1186/s13075-017-1293-1)
Supplement: Supplementary file 2 — Patient identification results. (DOCX 15 kb) [file 13075_2017_1293_MOESM2_ESM.docx]

**Patient Identification**

| **Step #** | **Criteria** | **Patient Counts** |
| --- | --- | --- |
| 1 | Patients with ≥1 day health plan enrollment (both medical and pharmacy) during the intake period (January 1, 2007 to April 30, 2014) | 32,679,057 |
| 2 | Patients with ≥1 fill/claim for TNFi agents^a^ (first Rx fill/medical claim as index date) during the intake period | 92,639 |
| 3 | Adult (≥18 years) patients as of index date | 89,297 |
| 4 | Patients with ≥2 outpatient (distinct service date) or ≥1 inpatient/ED medical claims for RA^b^ at any point during the study period (January 1, 2006 to April 30, 2015) | 42,222 |
| 5 | Patients with ≥12 months of health plan continuous enrollment pre-index date^c^ | 25,027 |
| 6 | Patients without any fills for any biologics^d^ over 12 months pre-index (biologic-naïve) | 15,021 |
| 7 | Patients with ≥12 months of health plan continuous enrollment post-index date | 11,667 |
| 8 | Excluding patients with ≥1 claim for any of the following conditions: comorbid psoriasis or psoriatic arthritis (696.xx), ankylosing spondylitis (720.0x), Crohn’s disease (555.xx), ulcerative colitis (556.xx) and juvenile chronic polyarthritis (714.3x), at any time during the study period | 7,797 |
| ED=emergency department; RA=rheumatoid arthritis; TNFi=tumor necrosis factor inhibitor  ^a^TNFi agents included adalimumab, certolizumab pegol, etanercept, golimumab, and infliximab  ^b^RA ICD-9-CM diagnosis codes: 714.0x, 714.1x, 714.2x  ^c^For all steps, continuous enrollment refers to patients with commercial, Medicare Advantage, or Medicare Supplemental plus Part D insurance plans during the indicated time frames  ^d^Biologics included all TNFi agents listed above, plus abatacept, anakinra, rituximab, tocilizumab, and tofacitinib citrate | | |
