# Supplementary material for: Costs associated with failure to respond to treatment among patients with rheumatoid arthritis initiating TNFi therapy: a retrospective claims analysis
Source: Arthritis Res Ther. 2017 May 15;19:92. doi: 10.1186/s13075-017-1293-1 (PMC5433023; doi:10.1186/s13075-017-1293-1)
Supplement: Supplementary file 3 — Patient characteristics and baseline HCRU/costs before matching. (DOCX 21 kb) [file 13075_2017_1293_MOESM3_ESM.docx]

**Baseline Demographic and Clinical Characteristics (Before Matching)**

|  | **Responders**  **(n=2,337)** | **Non-responders**  **(n=5,460)** | **p-Value^a^** | **Standardized Difference^b^** |
| --- | --- | --- | --- | --- |
| Female, n (%) | 1,655 (70.8%) | 4,203 (77.0) | <.001 | 0.14 |
| Age at index (years), mean (SD) | 52.3 (11.30) | 52.8 (12.65) | .101 | 0.04 |
| Geographic region, n (%) |  |  |  |  |
| Northeast | 348 (14.9%) | 806 (14.8) | .883 | 0.00 |
| South | 689 (29.5%) | 1,372 (25.1) | <.001 | 0.10 |
| Midwest | 731 (31.3%) | 1,724 (31.6) | .797 | 0.01 |
| West | 453 (19.4%) | 1,295 (23.7) | <.001 | 0.11 |
| Unknown | 116 (5.0%) | 263 (4.8) | .782 | 0.01 |
| Health plan type, n (%) |  |  |  |  |
| HMO | 590 (25.2%) | 1,271 (23.3) | .062 | 0.05 |
| PPO | 1,604 (68.6%) | 3,878 (71.0) | .034 | 0.05 |
| CDHP | 143 (6.1%) | 311 (5.7) | .465 | 0.02 |
| Any Medicare plan (Medicare Advantage or Medicare Supplemental plus Part D), n (%) | 182 (7.8%) | 758 (13.9) | <.001 | 0.20 |
| TNFi agent on index fill, n (%) |  |  |  |  |
| Adalimumab | 586 (25.1%) | 1,313 (24.0) | .333 | 0.02 |
| Certolizumab pegol | 24 (1.0%) | 100 (1.8) | .009 | 0.07 |
| Etanercept | 1,418 (60.7%) | 2,770 (50.7) | <.001 | 0.20 |
| Golimumab | 56 (2.4%) | 114 (2.1) | .393 | 0.02 |
| Infliximab | 253 (10.8%) | 1,163 (21.3) | <.001 | 0.29 |
| Prescribing physician specialty on index TNFi claim, n (%) |  |  |  |  |
| Rheumatology | 1,872 (80.1%) | 3,775 (69.1%) | <0.001 | 0.25 |
| PCP^c^ | 35 (1.5%) | 97 (1.8%) | 0.382 | 0.02 |
| Other | 47 (2.0%) | 103 (1.9%) | 0.713 | 0.01 |
| Unknown | 383 (16.4%) | 1,485 (27.2%) | <0.001 | 0.26 |
| QCI, mean (SD) | 1.5 (1.03) | 1.7 (1.28) | <.001 | 0.19 |
| CIRAS, mean (SD) | 6.6 (1.73) | 6.4 (1.83) | .001 | 0.08 |
| Targeted comorbidites of interest, n (%) |  |  |  |  |
| Chronic respiratory/pulmonary conditions | 305 (13.1%) | 1,046 (19.2%) | <.001 | 0.17 |
| CVD | 197 (8.4%) | 602 (11.0%) | 0.001 | 0.08 |
| Diabetes | 280 (12.0%) | 781 (14.3%) | .006 | 0.07 |
| Dyslipidemia | 789 (33.8%) | 1,948 (35.7%) | .104 | 0.04 |
| Fibromyalgia | 316 (13.5%) | 1,008 (18.5%) | <.001 | 0.14 |
| Fragility fractures (closed) | 34 (1.5%) | 127 (2.3%) | .013 | 0.06 |
| GI ulcer | 23 (1.0%) | 93 (1.7%) | .016 | 0.06 |
| Hypertension | 809 (34.6%) | 2,219 (40.6%) | <.001 | 0.13 |
| Low-back pain | 461 (19.7%) | 1,568 (28.7%) | <.001 | 0.21 |
| Mental health issues | 476 (20.4%) | 1,553 (28.4%) | <.001 | 0.19 |
| Osteoarthritis | 892 (38.2%) | 2,321 (42.5%) | <.001 | 0.09 |
| Osteoporosis | 231 (9.9%) | 785 (14.4%) | <.001 | 0.14 |
| CVD includes ACS (MI and unstable angina), CHD with or without history of MI, Ischemic stroke/TIA, PAD, and ventricular arrhythmia..  ACS=acute coronary syndrome; CDHP=consumer driven health plan; CHD=coronary heart disease; CIRAS=claims-based index for RA severity; CVD=cardiovascular disease; GI=gastrointestinal; HMO=health maintenance organization; MI=myocardial infarction; PAD=peripheral artery disease; PCP=primary care physician; PPO=preferred provider organization; QCI=Quan-Charlson Index; SD=standard deviation; TIA=transient ischemic attack; TNFi=tumor necrosis factor inhibitor  ^a^χ^2^ tests were used to determine any statistical difference across categorical variables; t-tests were used for continuous variables.  ^b^Standardized difference = difference in means or proportions divided by standard error, in absolute value.  ^c^PCP includes family/general practice and internal medicine. | | | | |

**Baseline All-Cause Healthcare Resource Utilization and Cost Characteristics (Before Matching)**

|  | **Responders**  **(n=2,337)** | **Non-responders**  **(n=5,460)** | **p-Value^a^** | **Standardized Difference^b^** |
| --- | --- | --- | --- | --- |
| Inpatient hospitalization, n (%) with ≥1 visit | 197 (8.4%) | 733 (13.4%) | <0.001 | 0.16 |
| ED encounters, n (%) with ≥1 visit | 374 (16.0%) | 1,126 (20.6%) | <0.001 | 0.12 |
| Outpatient visits, n (%) with ≥1 visit | 2,332 (99.8%) | 5,438 (99.6%) | 0.193 | 0.03 |
| Rheumatologist office visits, n (%) | 1,692 (72.4%) | 3,816 (69.9%) | 0.026 | 0.06 |
| Physical/occupational therapy visits, n (%) | 506 (21.7%) | 1,339 (24.5%) | 0.006 | 0.07 |
| Pharmacy fills, n (%) with ≥1 fill | 2,298 (98.3%) | 5,337 (97.7%) | 0.098 | 0.04 |
| Oral glucocorticoids | 1,579 (67.6%) | 3,882 (71.1%) | 0.002 | 0.08 |
| Antihypertensives | 887 (38.0%) | 2,384 (43.7%) | <0.001 | 0.12 |
| Antidiabetics | 206 (8.8%) | 537 (9.8%) | 0.160 | 0.04 |
| Antihyperlipidemics | 512 (21.9%) | 1,265 (23.2%) | 0.224 | 0.03 |
| Antidepressives | 537 (23.0%) | 1,785 (32.7%) | <0.001 | 0.22 |
| Pain medications | 1,853 (79.3%) | 4,399 (80.6%) | 0.195 | 0.03 |
| Number of pharmacy fills per patient, mean (SD) | 40.7 (28.11) | 44.8 (32.75) | <.0001 | 0.14 |
| csDMARDs, n (%) with ≥1 fill | 2,135 (91.4%) | 4,726 (86.6%) | <0.001 | 0.15 |
| Hydroxychloroquine | 701 (30.0%) | 1,538 (28.2%) | 0.102 | 0.04 |
| Leflunomide | 301 (12.9%) | 850 (15.6%) | 0.002 | 0.08 |
| Methotrexate | 1,849 (79.1%) | 3,905 (71.5%) | <0.001 | 0.18 |
| Minocycline | 27 (1.2%) | 83 (1.5%) | 0.211 | 0.03 |
| Sulfasalazine | 266 (11.4%) | 606 (11.1%) | 0.716 | 0.01 |
| Total medical costs, $ per person, mean (SD) | 6,819 (14,807) | 9,829 (25,917) | <0.001 | 0.14 |
| Inpatient costs, $ per person, mean (SD) | 1,927 (10,790) | 3,305 (21,993) | 0.004 | 0.08 |
| ED costs, $ per person, mean (SD) | 242 (938) | 393 (1,431) | <0.001 | 0.13 |
| Outpatient costs, $ per person, mean (SD) | 4,638 (7,152) | 6,063 (10,306) | <0.001 | 0.16 |
| Total pharmacy costs, $ per person, mean (SD) | 1,639 (2,433) | 2,107 (3,865) | <.0001 | 0.15 |
| csDMARD=conventional synthetic disease modifying antirheumatic drugs; ED=emergency department; SD=standard deviation;  ^a^χ^2^ tests were used to determine any statistical difference across categorical variables; t-tests were used for continuous variables  ^b^Standardized difference = difference in means or proportions divided by standard error, in absolute value. | | | | |
